# Lin28 promoting the protective effect of PMSCs on hepatic ischaemia–reperfusion injury by regulating glucose metabolism

**DOI:** 10.1111/jcmm.17739

**Published:** 2023-04-02

**Authors:** Xi Zhou, Junbo Li, Jin Wang, Huifang Yang, Xiaoyun Xie, Zhishui Chen, Bo Zhang

**Affiliations:** ^1^ Institute of Organ Transplantation, Tongji Hospital, Tongji Medical College Huazhong University of Science and Technology Wuhan China; ^2^ Key Laboratory of Organ Transplantation, Ministry of Education, Ministry of Public Health Chinese Academy of Medical Sciences Wuhan China; ^3^ NHC Key Laboratory of Organ Transplantation Wuhan China; ^4^ Key Laboratory of Organ Transplantation Chinese Academy of Medical Sciences Wuhan China; ^5^ Department of Interventional and Vascular Surgery Tenth People's Hospital of Tongji University No. 301 Middle Yan Chang Road Shanghai 200072 China; ^6^ Institute of Interventional and Vascular Surgery Tongji University No. 301 Middle Yan Chang Road Shanghai 200072 China

**Keywords:** glucose metabolism, human placental mesenchymal stem cells, lactate dehydrogenase a, liver ischaemia–reperfusion injury, PI3K‐Akt pathway

## Abstract

Human placental mesenchymal stem cells (PMSCs) can prevent liver ischaemia–reperfusion injury (LIRI). However, their therapeutic effects are limited. Therefore, additional research is required to elucidate the mechanisms of PMSC‐mediated LIRI prevention and enhance the related therapeutic effects. This study aimed to examine the role of the Lin28 protein in the regulation of glucose metabolism in PMSCs. Further, it explored whether Lin28 could enhance the protective effects of PMSCs against LIRI and investigated the underlying mechanisms. Western blotting was performed to examine Lin28 expression in PMSCs under hypoxic conditions. A Lin28 overexpression construct was introduced into PMSCs, and the effect on glucose metabolism was examined using a glucose metabolism kit. Further, the expression of some proteins involved in glucose metabolism and the PI3K‐AKT pathway and the levels of microRNA Let‐7a–g were examined using western blots and real‐time quantitative PCR, respectively. To examine the relationship between Lin28 and the PI3K‐Akt pathway, the effects of AKT inhibitor treatment on the changes induced by Lin28 overexpression were examined. Subsequently, AML12 cells were co‐cultured with PMSCs to elucidate the mechanisms via which PMSCs prevent hypoxic injury in liver cells in vitro. Finally, C57BL/6J mice were used to establish a partial warm ischaemia–reperfusion model. The mice received intravenous injections containing PMSCs (control and Lin28‐overexpressing PMSCs). Finally, their serum transaminase levels and degree of liver injury were assessed using biochemical and histopathological methods, respectively. Lin28 was upregulated under hypoxic conditions in PMSCs. Lin28 exerted protective effects against hypoxia‐induced cell proliferation. Moreover, it increased the glycolytic capacity of PMSCs, allowing PMSCs to produce more energy under hypoxic conditions. Lin28 also activated the PI3K‐Akt signalling pathway under hypoxic conditions, and its effects were attenuated by AKT inhibition. Lin28 overexpression could protect cells against LIRI‐induced liver damage, inflammation and apoptosis and could also attenuate hypoxia‐induced hepatocyte injury. Lin28 enhances glucose metabolism under hypoxic conditions in PMSCs, thereby exerting protective effects against LIRI by activating the PI3K‐Akt signalling pathway. Our study is the first to report the potential of genetically modified PMSCs for LIRI treatment.

## 1 BACKGROUND

Liver ischaemia–reperfusion injury (LIRI) is an important clinical complication occurring in patients undergoing liver surgery (e.g. transplantation and hepatectomy) and in cases of shock.^1^ LIRI can cause primary graft dysfunction after transplantation, liver dysfunction after hepatectomy and biliary tract injury.^2^, ^3^ During liver surgery, hepatic blood vessels are often blocked, leading to ischaemia and hepatocyte death.^4^ In addition, once blood supply is restored, free radicals attack healthy hepatocytes. Together, these events lead to necrosis and apoptosis, causing inflammatory cell infiltration and the aggregation of inflammatory mediators, resulting in further liver damage.^5^, ^6^ Studies have shown that the damage caused by reperfusion is far greater than that caused by ischaemia.^7^ Given their clinical impact, the mechanisms underlying LIRI have been studied extensively. Surgical strategies that reduce the duration of liver ischaemia, clinical approaches that provide intraoperative and postoperative liver protection, and immunosuppressive drugs that prevent liver damage have helped in decreasing the incidence of LIRI.^8^, ^9^, ^10^ Mesenchymal stem cells (MSCs) are present at multiple sites in the body, including the bone marrow, cord blood and umbilical cord and placental tissue. In adults, these cells represent non‐haematopoietic stem cells with multidirectional potential. MSCs are mainly used during bone marrow transplantation to prevent graft versus host disease, improve tissue repair and provide anti‐inflammatory and immunomodulatory effects.^11^, ^12^, ^13^, ^14^ Placental MSCs (PMSCs) show better immunogenicity and immune regulation than other types of MSCs, because they act as negative immune regulators and can inhibit the body's immune response^15^ . These cells can restore the immune balance and inhibit the inflammation associated with injury in several organs. Therefore, these cells are used for kidney, brain, heart and liver treatment.^16^, ^17^, ^18^ However, the widespread clinical application of MSCs is hindered due to some challenges. These include the short survival duration of MSCs, number of organs to be protected, and injury to MSCs during ischaemia and reperfusion.^19^ Further, the molecular regulation of MSC survival under hypoxic conditions is unclear.

Cancerous cells typically gain energy from glycolysis instead of aerobic respiration. Owing to this switch, called the Warburg effect, cancer cells show unique mitochondrial utilization.^20^ Lin28 is an RNA‐binding protein required for early embryonic development, stem cell differentiation/reprogramming, tumorigenesis and metabolism.^21^, ^22^, ^23^ Studies have shown that Lin28 protein can protect various organs from ischaemia–reperfusion injury.^24^, ^25^ Studies have shown that the Lin28/Let‐7 pathway can regulate mammalian glucose metabolism and is itself intricately controlled.^26^ Another important intracellular pathway is the PI3K‐Akt signalling pathway, which regulates processes such as energy metabolism, growth and development and autophagy.^27^, ^28^ Studies have shown that this pathway is activated under anoxic conditions and enables the switch from aerobic respiration to glycolysis, allowing cells to produce the required energy.^29^ However, whether Lin28 can enhance metabolism in human PMSCs during hypoxia and protect hepatocytes against LIRI is unclear. Moreover, the role of the PI3‐Akt pathway in this process remains to be elucidated.

In the present study, a partial warm ischaemia–reperfusion injury mouse model and hypoxia‐treated hepatocyte model were used to examine whether Lin28 can increase the glycolytic capacity of PMSCs under hypoxic conditions and protect liver cells against LIRI. Further, the involvement of PI3‐Akt signalling in the effects of Lin28 on PMSC glycolysis was also explored.

## 2 METHODS

### 2.1 Animals

We purchased male C57BL/6J mice (6–8 weeks old) from Beijing Vital River Laboratory Animal Technology Co., Ltd. The animals were housed in the Animal Facility of Tongji Hospital, Tongji Medical College, Huazhong University of Science and Technology. All animal experiments were conducted after approval from the Institutional Animal Care and Use Committee of the Tongji Medical College, Huazhong University of Science and Technology. All procedures complied with the relevant guidelines and regulations of the Chinese Council on Animal Care.

### 2.2 Partial LIRI model

Male C57BL/6J mice (20–22 g) were randomly assigned to one of four groups: Sham; LIRI; LIRI + PMSC; and LIRI + PMSC‐Lin28. A partial LIRI model was developed using methods described in an earlier study.^30^ The mice in the sham group underwent the same operative procedure, without any blood vessel blocking. The LIRI + PMSC and LIRI + PMSC‐Lin28 groups received injections with PMSCs or PMSCs overexpressing Lin28 (PMSCs‐Lin28). The cells were injected into the portal vein (100 μL, 10^7^ cells/mL) 1 h prior to the induction of hepatic ischaemia. After 1 h of ischaemia and 6 h of reperfusion, mice were sacrificed, and serum and liver tissue samples were collected.

### 2.3 Serum biochemistry assay

Blood collected from mice was first chilled on ice (30 min) and then centrifuged at 8000 RPM (15 min). The supernatant was diluted as appropriate and a standard automatic analyser (Mindray BS‐200) was used to detect serum alanine transaminase (ALT) and aspartate transaminase (AST) levels.

### 2.4 Cell culture and hepatocyte hypoxia model

Human PMSCs (Shanghai Tongji University) and murine hepatocyte cells (AML12 cell line; ATCC®CRL‐2254TM) were cultured in Dulbecco's modified eagle medium/F‐12 (Hyclone) supplemented with 10% foetal bovine serum (Hyclone), 40 ng/mL dexamethasone (Sigma‐Aldrich, D4902), and ITS Liquid Media Supplement (Sigma‐Aldrich, I3146). The AML12 cells and PMSCs were placed in normoxic and anoxic environments, respectively, at 37°C. A humidified incubator containing 5% CO_2_ was used to maintain a normoxic environment. In contrast, a hypoxia incubator (Whitley H35 Hypoxystation) containing 5% CO_2_, 1% O_2_ and 94% N_2_ was used for maintaining an anoxic environment. To establish the ischemic and hypoxic cellular models, cells were cultured to a density of approximately 70%. Subsequently, the old medium was removed, and serum‐free medium was added; cells were incubated in anoxic conditions for 24 h. Additionally, cells in the inhibitor treatment group were treated with MK2206 (AKT inhibitor; Sellect, S1078; final concentration, 3 μM). Four treatment groups were established: PMSC, PMSC‐Lin28, PMSC + MK2206 2Hcl and PMSC‐Lin28 + MK2206. We maintained AML12 cells under hypoxic conditions for 24 h to construct a cellular model of ischaemia and hypoxia. The cells were divided into three groups: the AML12 group (AML12 cells alone), AML12 + PMSC group (AML12 cells co‐cultured with PMSCs) and AML12 + PMSC‐Lin28 group (AML12 cells co‐cultured with PMSCs‐Lin28).

### 2.5 Overexpression of Lin28

When cells reached a density of approximately 70%, they were cultured in OPTI‐MEM Reduced Serum Medium (Gibco) with a Lin28 overexpression lentivirus (10 μL/mL; with green fluorescence; obtained from Hanbio Biotechnology Co. Ltd.) for 72 h. Subsequently, fluorescence microscopy was used to detect transfection efficiency.

### 2.6 Cellular glucose metabolism assay

Glucose (Solarbio, BC2500), lactic acid (LA; Solarbio, BC2230), ATP (Solarbio, BC0300) and NADPH (Solarbio, BC1100) detection kits were used to detect intracellular glucose levels, LA levels, ATP levels, and the NADPH/NADP+ ratio, respectively. ECAR was detected using the Seahorse XF Glycolysis Stress Test Kit (Seahorse, 103,020–100).

### 2.7 Pathology

#### 2.7.1 Haematoxylin and eosin (H&E) staining

After 6 h of ischaemia, liver tissue was fixed in 4% paraformaldehyde. The tissue was dehydrated using an increasing ethanol concentration gradient and embedded in paraffin. Subsequently, 4‐μm‐thick sections were obtained and stained with Haematoxylin and Eosin. The stained samples were examined by two independent pathologists, who assessed hepatic necrosis based on Suzuki's scores.^31^

#### 2.7.2 TUNEL assay

A TUNEL Kit (Servicebio, G1501) was used based on manufacturer's instructions. First, tissue sections were deparaffinized using protease K. Subsequently, membranes were disrupted, the reaction solution was added and nuclei were stained with DAPI. Finally, the sections were imaged using an upright fluorescence microscope (NIKON ECLIPSE C1). The proportion of TUNEL‐positive cells was calculated using five randomly selected fields.

#### 2.7.3 Myeloperoxidase staining

Myeloperoxidase (MPO) immunohistochemistry was performed using mouse anti‐MPO (Servicebio, GB12224) and HRP‐labelled goat anti‐mpO (Servicebio, G23301) antibodies and the histochemical DAB chromogenic agent kit (Servicebio, GB12224). All procedures were performed based on manufacturer's instructions. The fixed tissue was first blocked with serum. Subsequently, the primary antibody, secondary antibody and developer were added. Finally, the nuclei were stained with DAPI. Then, the tissue was examined under a microscope, and images were captured and analysed. The haematoxylin‐stained nuclei appeared blue, whereas positive mPO expression was indicated by a brownish yellow colour.

### 2.8 Cell viability assay

The CCK‐8 kit (Dojindo) was used to detect cell viability. Before hypoxia treatment, 10,000 cells were planted in each well of a 96‐well plate, and three holes were made in each well. After 24 h of culture, the medium was replaced and 10 μL of the CCK‐8 reagent was added to each well (incubation, 37°C, 2 h). Finally, a microplate reader was used to measure the absorbance at 450 nm.

### 2.9 Flow cytometry analysis

An Annexin V‐FITC and propidium iodide staining kit (MULTISCIENCES) was used to examine cell apoptosis using manufacturer's instructions. Using a Flow Cytometer (BD FACSCelesta) and FlowJo software for data analysis, we measured the percentages of apoptotic cells.

### 2.10 qRT‐PCR

The Total RNA Rapid Extraction Kit (Fastagen) was used to isolate the total mRNA from PMSCs. The mRNA was then reverse transcribed using a high‐capacity cDNA reverse transcription kit (TAKARA) based on manufacturer's instructions. Target gene expression was evaluated with qRT‐PCR using a SYBR Green kit (TAKARA). StepOne software (Thermo Fisher Scientific) was used to analyse the data. For examining microRNA levels, the high‐capacity microRNA reverse transcription kit (TAKARA) was used; the other steps remained the same as those for mRNA analysis. *GADPH* and *U6* were chosen as internal controls for mRNA and microRNA, respectively. Each experiment was performed at least in triplicate. Primer sequences are shown in the table below (Table 1). Relative gene expression was calculated using the 2^−ΔΔC^_T_ method.

**TABLE 1:** Primer sequences.

Gene	RT primer	Forward primer	Reverse primer
Let‐7a	5′‐GTCGTATCCAGTGCAGGGTCCGAGGTATTCGCACTGGATACGACAACTAT‐3′	5′‐GCGCGTGAGGTAGTAGGTTGT‐3′	5′‐AGTGCAGGGTCCGAGGTATT‐3′
Let‐7b	5′‐GTCGTATCCAGTGCAGGGTCCGAGGTATTCGCACTGGATACGACAACCAC‐3′	5′‐GCGCGTGAGGTAGTAGGTTGT‐3′	5′‐AGTGCAGGGTCCGAGGTATT‐3′
Let‐7c	5′‐GTCGTATCCAGTGCAGGGTCCGAGGTATTCGCACTGGATACGACAACCAT‐3′	5′‐GCGCGTGAGGTAGTAGGTTGT‐3′	5′‐AGTGCAGGGTCCGAGGTATT‐3′
Let‐7d	5′‐GTCGTATCCAGTGCAGGGTCCGAGGTATTCGCACTGGATACGACAACTAT‐3′	5′‐GCGCGAGAGGTAGTAGGTTGC‐3′	5′‐AGTGCAGGGTCCGAGGTATT‐3′
Let‐7e	5′‐GTCGTATCCAGTGCAGGGTCCGAGGTATTCGCACTGGATACGACAACTAT‐3′	5′‐CGCGTGAGGTAGGAGGTTGT‐3′	5′‐AGTGCAGGGTCCGAGGTATT‐3′
Let‐7f	5′‐GTCGTATCCAGTGCAGGGTCCGAGGTATTCGCACTGGATACGACAACTAT‐3′	5′‐CGCGCGTGAGGTAGTAGATTGT‐3′	5′‐AGTGCAGGGTCCGAGGTATT‐3′
Let‐7 g	5′‐GTCGTATCCAGTGCAGGGTCCGAGGTATTCGCACTGGATACGACAACTGT‐3′	5′‐CGCGCGTGAGGTAGTAGTTTGT‐3′	5′‐AGTGCAGGGTCCGAGGTATT‐3′
U6	5′‐GTCGTATCCAGTGCAGGGTCCGAGGTATTCGCACTGGATACGACAAAATA‐3′	5′‐AGAGAAGATTAGCATGGCCCCTG‐3′	5′‐CAGTGCAGGGTCCGAGGT‐3′
PIK3CA	–	5′‐CCACGACCATCATCAGGTGAA‐3′	5′‐CCTCACGGAGGCATTCTAAAGT‐3′
LDHA	–	5′‐ATGGCAACTCTAAAGGATCAGC‐3′	5′‐CCAACCCCAACAACTGTAATCT‐3′
GAPDH	–	5′‐AGGTCGGTGTGAACGGATTTG‐3′	5′‐TGTAGACCATGTAGTTGAGGTCA‐3′

### 2.11 Western blot

IP lysis buffer (Beyotime) with protease inhibitors was used to extract the total protein from cell lysates or tissue sample. A microplate reader (BioTek) and BCA Protein Assay Kit (Beyotime) were used to measure the protein concentration. Proteins (80 μg) were separated using 10% SDS‐PAGE and transferred onto polyvinylidene fluoride membranes (Millipore, 0.22 μm). The membranes were blocked with 5% skim milk in TBST and then incubated with primary antibodies for 16 h. Antibodies against β‐catenin (Abcam, ab32572), lactate dehydrogenase A (LDHA; Abcam, ab52488), AKT (Abcam, ab179463), pAKT (ser473), Lin28 (Abcam, ab191881), total OXPHOS (Abcam, ab110431), Hexokinase I (HEX I; Abcam, ab150423), Hexokinase II (HEX II; Abcam, ab209847), M2‐pyruvate kinase (PKM2; Abcam, ab85555), 3‐phosphoinositide dependent kinase‐1 (PDK1; Abcam, ab202468) and 6‐phosphofructo‐2‐kinase/fructose‐2,6‐bisphosphatase 2 (Abcam, ab234865) were used. Subsequently, the membranes were blocked with horseradish peroxidase‐conjugated goat anti‐rabbit IgG H&L or goat anti‐mouse IgG H&L antibody (Abcam, ab6721 and ab6789) at room temperature (20–25°C) for 1 h. The ECL reagent (Beyotime) was used to detect protein bands. The Chemiluminescence Imaging System (GeneGnome) and Image‐Pro Plus software (Media Cybernetics) were used for protein quantification. Proteins levels were normalized based on β‐catenin levels, and all experiments were performed at least thrice.

### 2.12 Statistical analysis

All statistical analyses were performed using Prism software (GraphPad, v8.0.2). All data were expressed as the mean ± standard error of the mean. One‐way anova was used to compare multiples groups, whereas unpaired *t*‐tests were used to compare two groups. *p*‐values < 0.05 were considered statistically significant.

## 3 RESULTS

### 3.1 Lin28 affects glucose metabolism levels in PMSCs

**FIGURE 1:**
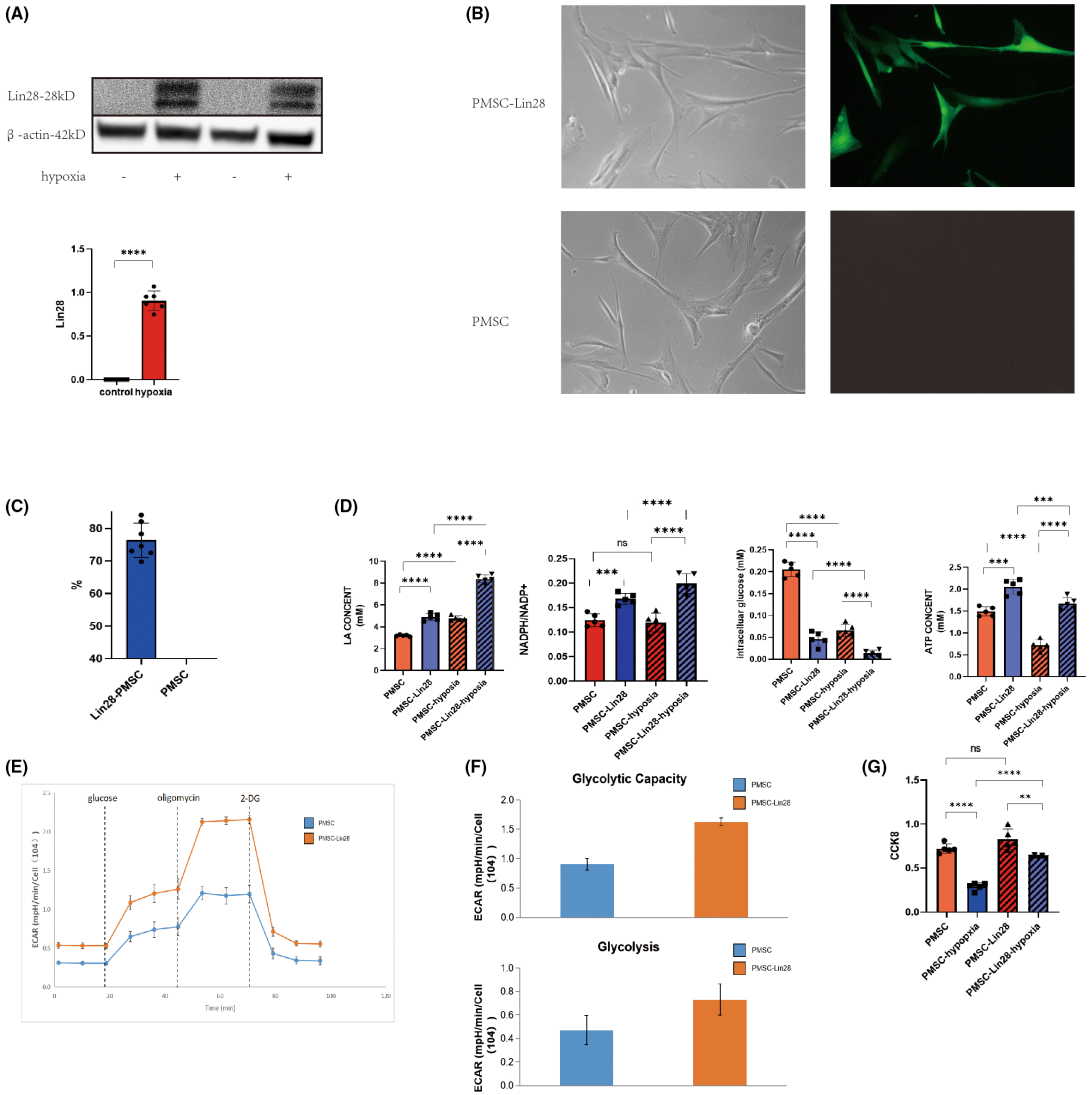
Lin28 enhance the glycolytic ability of PMSCs. (A) The protein levels of Lin28 in PMSCs after hypoxia were analyzed using western blots. (B) PMSCs were transfected with lentiviral constructs for green fluorescence‐tagged Lin28 and observed using fluorescence microscopy. (C) The proportion of green fluorescent cells (transfection ratio) was calculated by randomly selecting different fields. (D) Lactic acid (LA), intracellular glucose, NADP+/NADPH, and ATP levels were detected under normoxic conditions in PMSCs and PMSCs overexpressing LIN28. (E) Lactic acid (LA), intracellular glucose, NADP+/NADPH, and ATP levels were detected under hypoxic conditions in PMSCs and PMSCs overexpressing LIN28. (F) Glucose metabolism in PMSC and PMSCs‐Lin28 was measured using CAR, and glucose, oligomycin (ATP synthase inhibitor), and 2‐Deoxy‐d‐glucose (2‐dg) were added sequentially. (G) Basal glucose metabolism levels and the glycolytic ability of cells were calculated using ECAR, and significant differences were observed. ***p < 0.001; ****p < 0.0001.

A PMSC hypoxia model was used to examine the expression of Lin28 in PMSCs under hypoxic conditions. We found that hypoxia obviously increased Lin28 expression in PMSCs (Figure 1A). The Lin28 overexpression lentivirus showed a 76.36 ± 2.002% transfection efficiency in PMSCs (Figure 1B,C). On examining several biochemical indicators of glycolysis, we found that PMSCs transfected with Lin28 had a higher LA level, NADPH/NADP+ ratio and ATP content than non‐transfected cells. These cells also had lower intracellular glucose levels. Although these differences were also observed in normoxic environments, they were more obvious in anoxic environments (Figure 1D). Consistent with these findings, we observed an increase in the basal glucose metabolism and glycolytic capacity of PMSCs after Lin28 overexpression, indicating that Lin28 promoted glycolysis in PMSCs (Figure 1E,F). A CCK‐8 assay demonstrated that the proliferation of PMSCs was significantly reduced under hypoxia. Moreover, the proliferation of Lin28‐PMSCs was also significantly reduced (Figure 1G). These results indicated that the overexpression of Lin28 under hypoxic conditions could promote anaerobic glycolysis in PMSCs, allowing them to produce more energy under hypoxia and maintain cellular function.

### 3.2 Lin28 can regulate proteins related to glucose metabolism in PMSCs

**FIGURE 2:**
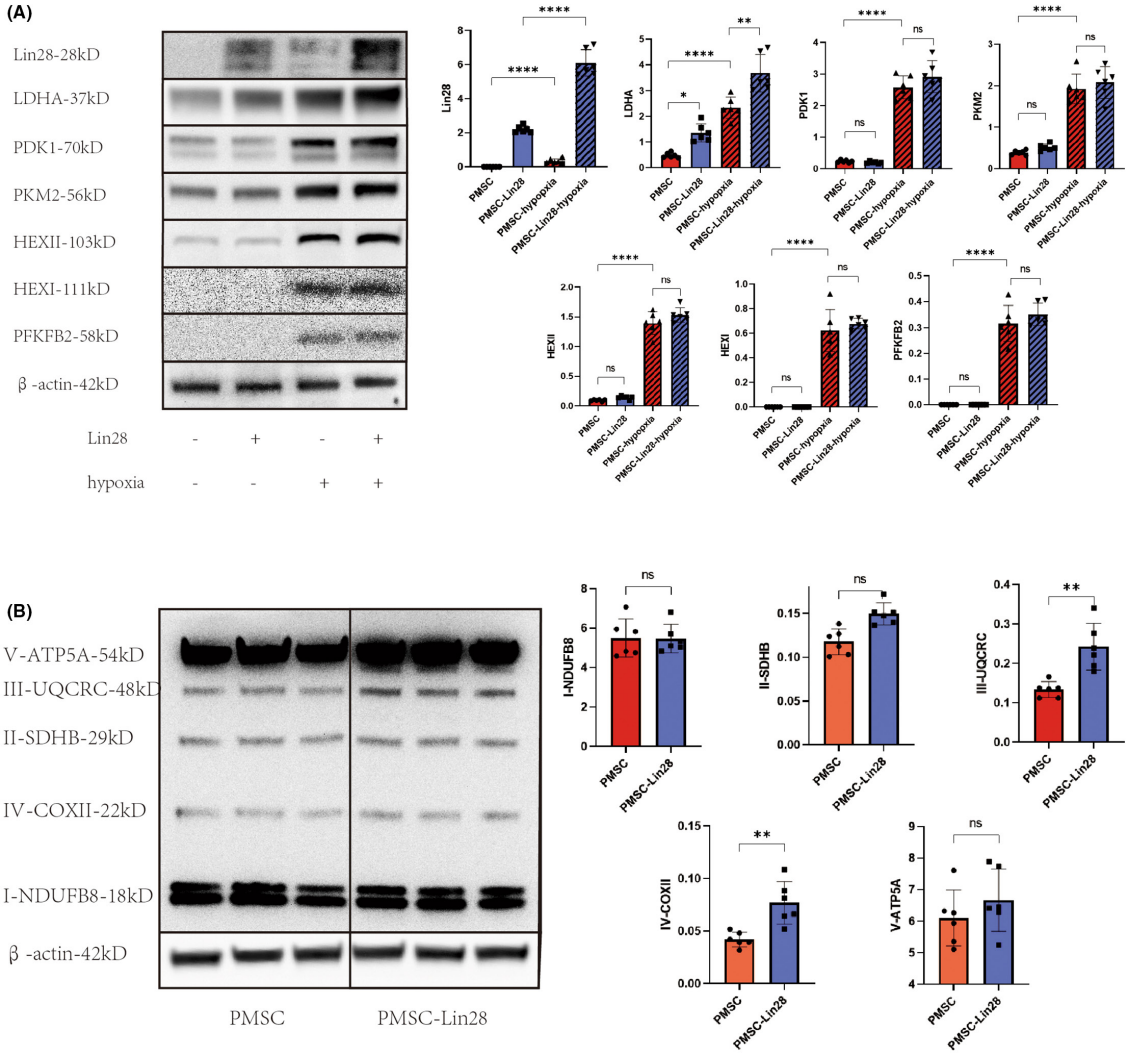
Expression of glucose metabolism‐related proteins in PMSCs and PMSCs‐Lin28 under normoxic and hypoxic conditions. (A) The protein expression of Lin28, lactate dehydrogenase A (LDHA), 3‐phosphoinositide dependent kinase‐1 (PDK1), M2‐pyruvate kinase (PKM2), Hexokinase I and II (HEX I and HEX 2), and 6‐phosphofructo‐2‐kinase/fructose‐2,6‐biphosphatase 2 (PFKFB2) analyzed using western blots. (B) The expression of proteins involved in oxidative phosphorylation, including NDUFB8 (18 kD), SDHB (29 kD), UQCRC (48 kD), COXII (22 kD), and ATP5A (54 kD) analyzed using western blots. ns, *p* > 0.05; *p < 0.05; **p < 0.01; ***p < 0.001; ****p < 0.0001.

In order to understand the pathways through which Lin28 increases the glycolytic capacity of PMSCs, we examined the levels of glycolysis‐related proteins in these cells under normoxic and hypoxic conditions. The proteins LDHA, PDK1, PKM2, HEX I, HEX II and PFKFB2 – which are enzymes involved in the regulation of glycolysis – were upregulated during hypoxia. However, these proteins, except LDHA, showed no significant difference in expression in PMSCs overexpressing Lin28 (Figure 2A). The mitochondrial proteins UQCRC and COXII were upregulated in PMSCs overexpressing Lin28; however, ATP5A, SDHB, and NDUFB8 showed no changes in expression (Figure 2B). Therefore, we speculated that Lin28 may regulate glucose metabolism in PMSCs via LDHA.

### 3.3 Lin28 regulates LDHA via the Let7‐PI3K‐Akt pathway

**FIGURE 3:**
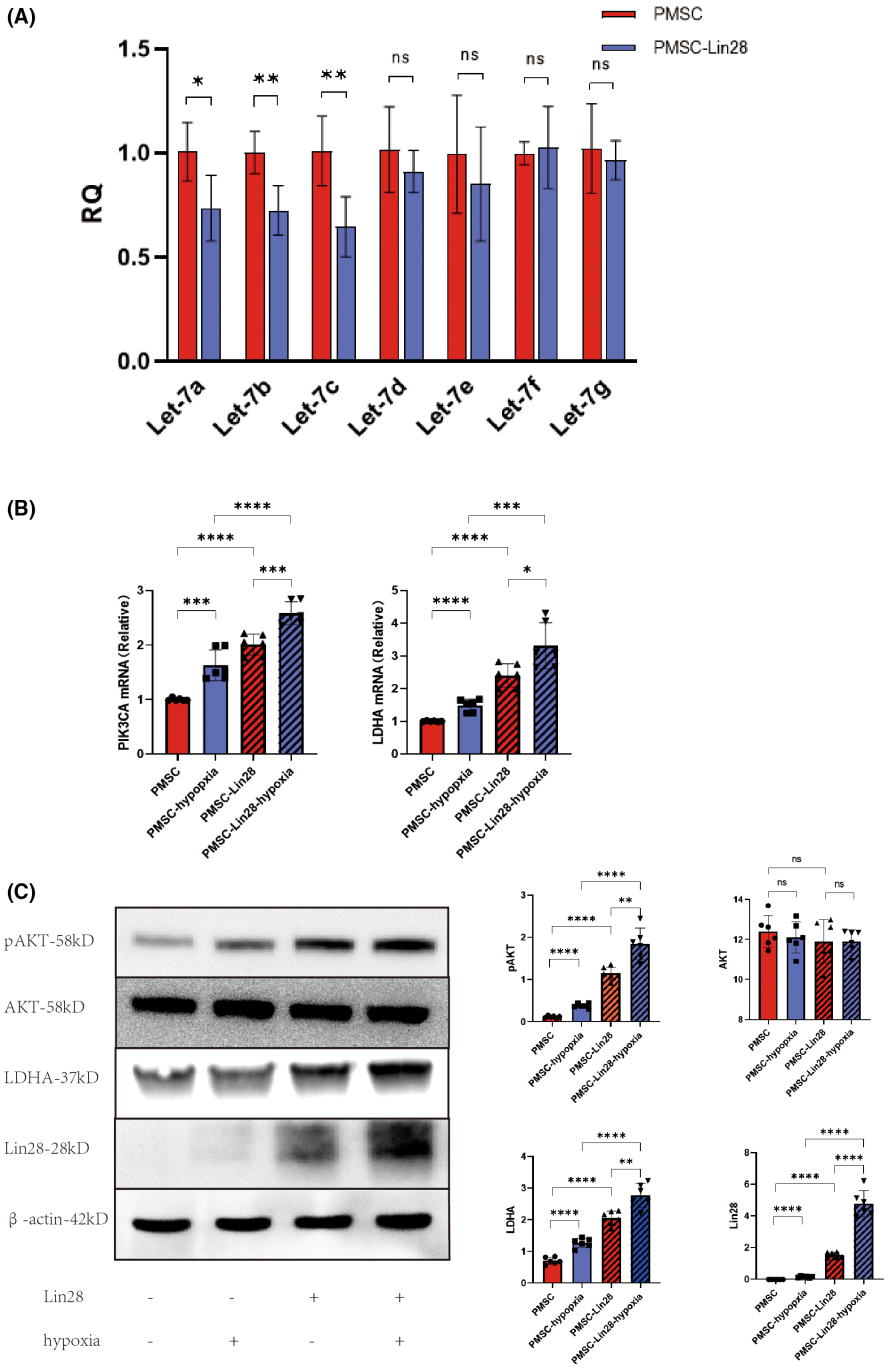
Lin28 regulates LDHA through the Let7‐PI3K‐Akt pathway. (A) The microRNA levels of Let‐7 family members were measured using real‐time polymerase chain reaction. (B) The mRNA levels of PI3K and LDHA were measured using RT‐PCR. (C) The protein expression of pAKT (ser 473), AKT, LDHA, and Lin28 in PMSCs and PMSCs‐Lin28 under normoxic and hypoxic conditions was analyzed using western blotting. ns, *p* > 0.05; *p < 0.05; **p < 0.01; ***p < 0.001; ****p < 0.0001.

Lin28 can downregulate microRNAs from the Let7 family. Using qRT‐PCR, we found that the levels of microRNA Let7a, Let7b and Let7c were dramatically lower in the PMSC‐Lin28 group than in the PMSC group. However, microRNA Let7d, Let7e, Let7f and Let7g showed no significant difference in expression (Figure 3A). Previous studies have confirmed that a reduction in Let7c levels leads to the activation of PI3K and the PI3K‐Akt pathway, altering the expression of LDHA. Thus, the mRNA levels of *PI3K* and *LDHA* were quantified in PMSCs and PMSCs‐Lin28 under normoxic and hypoxic conditions using qRT‐PCR. Moreover, the protein expression of AKT, pAKT (ser473), Lin28, and LDHA was detected using western blots. The transcriptional levels of *PI3K* and *LDHA* were higher in the PMSCs‐Lin28 group than in the PMSCs group. These levels were also higher under hypoxic conditions than under normoxic conditions (Figure 3B). The protein levels of pAKT (ser473) and LDHA showed similar trends. However, the protein expression of AKT was comparable among all groups (Figure 3C). Based on these results, we speculated that Lin28 downregulates Let7c by interacting with the microRNA. This results in PI3K upregulation, activating the PI3K‐Akt pathway and promoting AKT phosphorylation. Such changes lead to increased LDHA expression, promoting the switch from aerobic oxidation to anaerobic glycolysis in PMSCs.

### 3.4 Inhibitors of the PI3K‐Akt pathway weaken the effect of Lin28 on glucose metabolism

**FIGURE 4:**
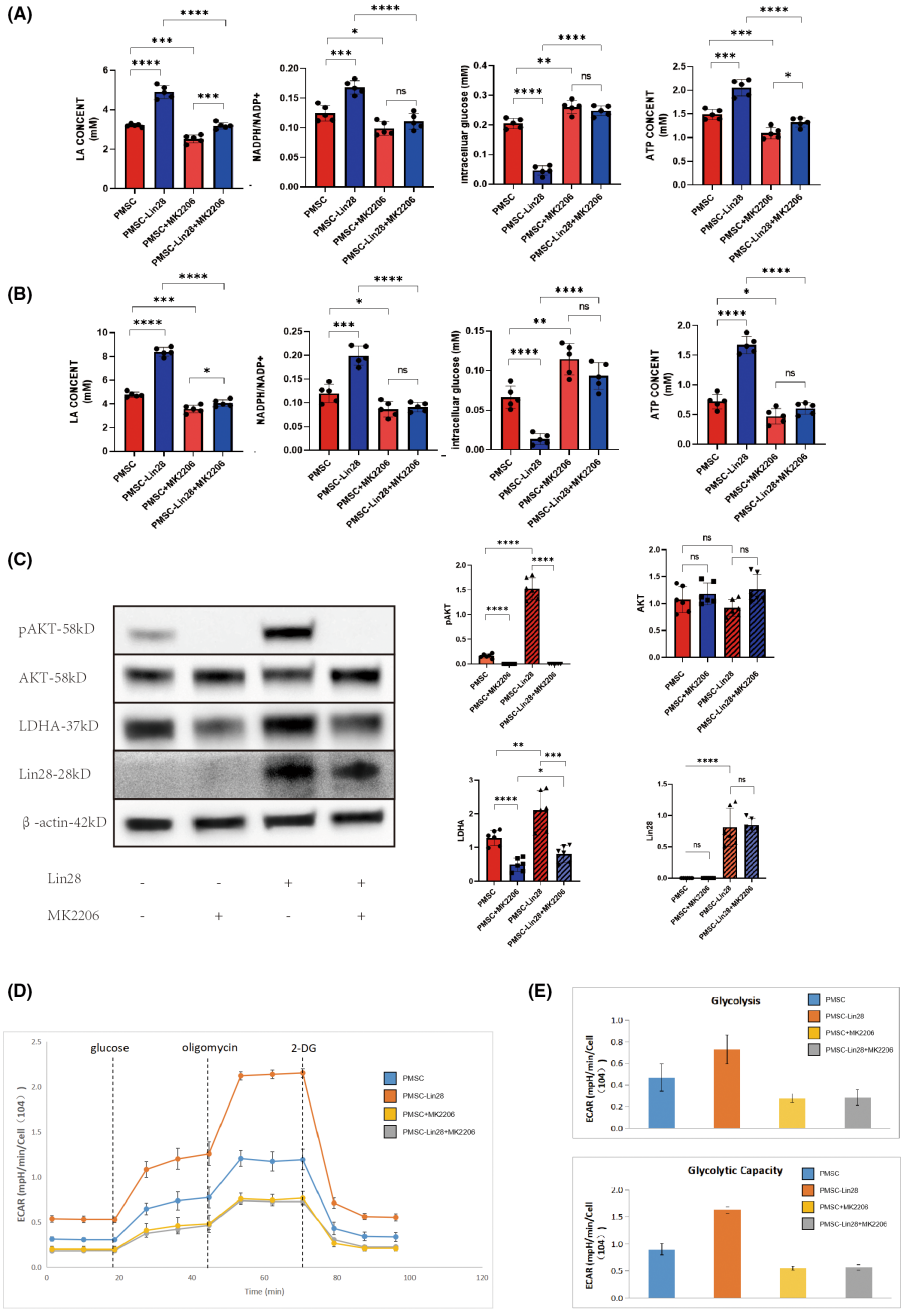
Effect of Lin28 on glucose metabolism in PMSCs after treatment with MK2206, an inhibitor of the PI3K‐Akt pathway. (A) Lactic acid (LA), intracellular glucose, NADP+/NADPH, and ATP levels detected in PMSCs and PMSCs‐LIN28 treated with MK2206 under normoxic conditions. (B) Lactic acid (LA), intracellular glucose, NADP+/NADPH, and ATP levels detected in PMSCs and PMSCs‐LIN28 treated with MK2206 under hypoxic conditions. (C) The protein expression of pAKT (ser 473), AKT, LDHA, and Lin28 was examined in PMSCs and PMSCs‐Lin28 treated with MK2206 under normoxic and hypoxic conditions using western blot. (D) Glucose metabolism was examined in PMSCs and PMSCs‐Lin28 treated with MK2206 using CAR, and glucose, oligomycin (ATP synthase inhibitor), and 2‐Deoxy‐d‐glucose (2‐dg) were added sequentially. (E) Basal glucose metabolism levels and the glycolysis ability of PMSCs and PMSCs‐Lin28 were calculated using ECAR, and significant differences were observed. ns, *p* > 0.05; *p < 0.01; **p < 0.01;***p < 0.001; ****p < 0.0001.

In order to verify that Lin28 regulates glucose metabolism via the PI3K‐Akt pathway, we treated PMSCs with the AKT phosphorylation inhibitor MK2206 in vitro. After treatment with MK2206, there was a decrease in intracellular LA levels, the NADPH/NADP+ ratio, and the ATP content in Lin28‐overexpressing PMSCs. Moreover, the intracellular glucose content increased significantly. Interestingly, these effects were observed under both normoxic and anoxic conditions (Figure 4A,B). On examining protein expression, we found that cells from the PMSC + MK2206 and PMSC‐Lin28 + MK2206 groups had lower levels of pAKT (ser473) and LDHA than those from the PMSC and PMSC‐Lin28 groups. In contrast, the levels of AKT and Lin28 were similar between the PMSC‐Lin28 + MK2206 and PMSC‐Lin28 groups and the PMSC + MK2206 and PMSC groups (Figure 4C). Furthermore, ECAR analysis showed that MK2206 treatment reduced basal glucose metabolism and glycolysis in PMSCs overexpressing Lin28 (Figure 4D,E). Hence, we concluded that Lin28 enhances the viability of PMSCs in anoxic environments by activating the PI3K‐Akt pathway.

### 3.5 Lin28 overexpression in PMSCs enhances their protective effects against ischaemia–reperfusion‐induced apoptosis in AML12 cells

**FIGURE 5:**
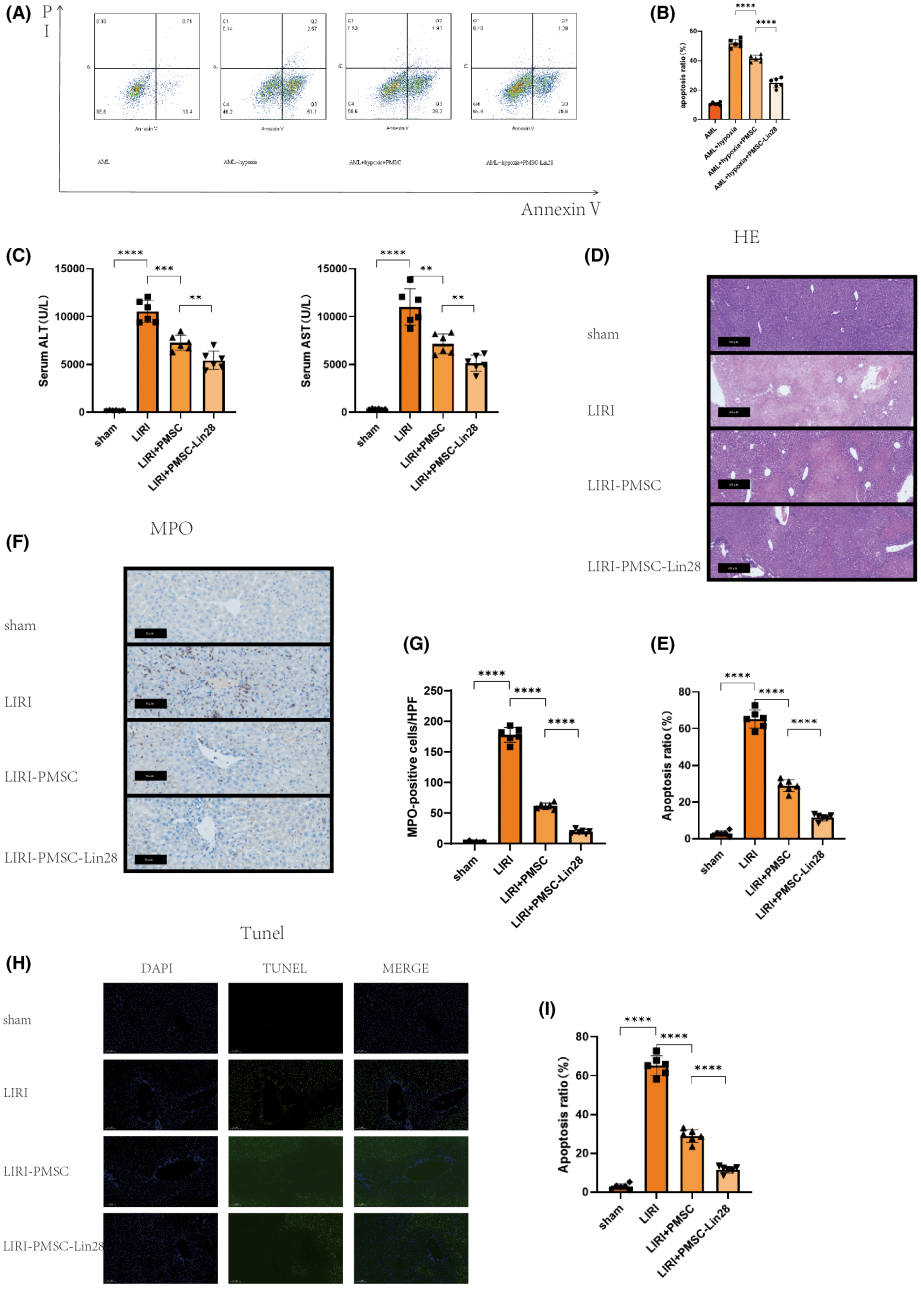
Lin28 enhanced the protective effect of PMSC against LIRI in vitro and in vivo. (A, B) Apoptosis rates examined with Annexin V/PI staining. (C) Serum ALT and AST levels were examined in mice after 6 h of reperfusion. (D) Hematoxylin and eosin‐stained liver tissues (×100). Scale bar = 100 μm. (E) Extent of liver injury graded based on Suzuki's scores. (F) MPO staining of liver sections from mice (×200). Scale bar = 50 μm. (G) Quantification of MPO‐positive cells. (H) TUNEL assay of the liver sections from mice (×200). Scale bar = 100 μm. (I) Quantification of TUNEL‐positive cells (×200). LIRI, liver ischemia–reperfusion injury; MPO, myeloperoxidase; PI, propidium iodide. All numerical data were obtained from *n* = 6 mice per group. **p < 0.01; ***p < 0.001; ****p < 0.0001.

After 24 h of anoxic culture, as mentioned above, the cells were cultured in a normoxic incubator containing 5% CO_2_ for 1 h. The cells were then harvested and analysed using flow cytometry. The apoptosis rate was found to be markedly lower in the AML12 + PMSC and AML12 + PMSC‐Lin28 groups than in the AML12 group. Furthermore, this value was much lower in the AML12 + PMSC‐Lin28 group than in the AML12 + PMSC group (Figure 5A,B). This indicated that Lin28 enhances the protective effects of PMSCs against apoptosis in the AML12 hypoxia model.

### 3.6 Lin28 overexpression in PMSCs enhances their protective effects against LIRI in mice

We examined the effects of treatment with Lin28‐overexpressing PMSCs in a mouse model of LIRI using biochemical and histological examinations. Serum ALT and AST levels in the LIRI group were significantly higher than those in the sham operation group. However, this effect was attenuated in the LIRI + PMSC group and even more significantly so in the LIRI + PMSC‐Lin28 group (Figure 5C). Similarly, mice in the LIRI group developed severe liver injury, with a significant increase in the area of necrotic liver tissue and Suzuki score. In contrast, the degree of liver damage was significantly lower in the LIRI + PMSC and LIRI + PMSC‐Lin28 groups, and especially in the LIRI + PMSC‐Lin28 group (Figure 5D,E). These observations suggested that the overexpression of Lin28 could increase the protective effects of PMSCs against LIRI. Given that the inflammatory response is also an important factor in LIRI, we examined inflammation‐related parameters such as the neutrophil infiltration ratio using MPO staining. In the LIRI + PMSC‐Lin28 and LIRI + PMSC groups, neutrophilic infiltration was reduced, with the effect being more obvious in the LIRI + PMSC‐Lin28 group (Figure 5F,G). This indicated that Lin28 overexpression could increase the effectiveness of PMSCs in reducing the inflammatory response during LIRI. In addition, the protective effect of PMSCs was also examined using TUNEL staining. As shown in the figure, the LIRI group had the most TUNEL‐positive cells. After PMSC treatment and especially after PMSC‐Lin28 treatment, the number of TUNEL‐positive cells decreased (Figure 5H,I). Hence, we concluded that Lin28 overexpression could improve the protective effects of PMSCs against hepatocyte apoptosis.

## 4 DISCUSSION

Orthotopic liver transplantation is now emerging as the frontrunner for the treatment of end‐stage liver disease. However, LIRI remains a key and common cause of postoperative hepatic impairments, inflammatory infiltration, and apoptosis. More importantly, its severity is tightly linked to postoperative recovery.^32^, ^33^ Many studies have focused on the treatment of LIRI.^34^, ^35^ MSCs are considered to be effective in reducing LIRI and consequently protecting liver function.^36^, ^37^, ^38^ The findings of the present study also prove their effectiveness. However, the mechanism by which MSCs protect the body against LIRI is unclear. Currently, it is hypothesized that these effects are related to the inhibition of mitochondrial reactive oxygen species overproduction. The improvement of mitochondrial function,^39^ regulation of miRNA,^40^ and regulation of immune cells^41^ by extracellular vesicles are also thought to play a role. Changes in macrophage activation can prevent inflammation.^42^ PMSCs, which express higher levels of several immunomodulatory and pro‐angiogenic cytokines than other MSCs, are widely used for the prevention of ischaemia–reperfusion injury.^43^ However, during liver ischaemia, MSCs are themselves exposed to ischaemia and hypoxia. Currently, the effect of these conditions on the cellular functions of MSCs and the mechanisms underlying these effects are unclear.

In the present study, we first constructed a hypoxia model in PMSCs and observed an increase in Lin28 protein levels. Subsequently, we transfected Lin28 into PMSCs using a lentivirus. We showed that Lin28 can increase anaerobic fermentation in PMSCs in a hypoxic environment and increase energy production in an anaerobic state, enabling cell survival. Next, we sought to identify the pathways that mediated the effects of Lin28. Hence, we examined several key regulators of glycolysis, including LDHA, PDK1, PKM2, HEX I, HEX II and PFKFB2.^44^, ^45^ Our results confirmed that these enzymes were significantly upregulated under hypoxic conditions. However, only LDHA showed a significant increase in PMSCs overexpressing Lin28. Meanwhile, Lin28 overexpression had little effect on the expression of mitochondrial complex proteins. Together, these results indicated that LDHA may be a key mediator of the effects of Lin28 on anaerobic fermentation in PMSCs.

Next, we studied the effects of Lin28 on LDHA. Lin28 can downregulate members of the microRNA Let‐7 family by binding to the microRNA Let‐7 precursor.^46^, ^47^ Our experiments also verified these findings. Further, the increased expression of LDHA under hypoxia allows cells to switch from oxidative phosphorylation to anaerobic glycolysis. This switch mainly occurs through the activation of the PI3K‐Akt pathway.^48^, ^49^ Our experiments, as expected, showed that the PI3K‐Lin28 pathway was activated in PMSCs after Lin28 transfection. To verify that the effects of Lin28 on LDHA expression were mediated by this pathway, we used the AKT inhibitor MK2206 2Hcl, which can effectively inhibit the phosphorylation of Ser473 in AKT.^50^ After treatment with this inhibitor, the positive effects of Lin28 overexpression on aerobic glycolysis, energy generation under anoxic conditions, and LDHA levels were significantly inhibited, despite Lin28 expression being unaffected. This demonstrated that AKT inhibitors can attenuate the Lin28‐mediated regulation of LDHA without affecting Lin28 levels. The findings suggested that Lin28 regulates LDHA via the PI3K‐Akt pathway, thereby enhancing anaerobic glycolysis in PMSCs. Therefore, Lin28 overexpression could increase energy production in PMSCs even in an anoxic environment. Hence, we speculated that Lin28 overexpression could enhance the protective effects of PMSCs against LIRI.

We designed an in vitro cellular model of hypoxia using AML12 cells co‐cultured with PMSCs. Then, we examined whether Lin28‐overexpressing PMSCs were more effective in protecting hepatocytes from hypoxia. Lin28‐overexpressing PMSCs significantly reduced the apoptosis rate in AML12 cells after anoxia, conferring a better protective effect than normal PMSCs. To confirm this effect in vivo, we constructed a mouse LIRI model. Studies have shown that most MSCs disappear around 2 h after caudal intravenous infusion. Only a few surviving MSCs reach the liver, resulting in failed protection against LIRI.^51^ This also explains why several studies on LIRI have focused on MSC‐derived extracellular vesicles instead of MSCs themselves. If MSCs are infused after reperfusion, they may be unable to prevent the hepatic damage caused by ischaemia. Hence, we injected PMSCs into the portal vein 1 h before ischaemia, allowing MSCs to accumulate in the liver and protecting stem cells from ischaemia–reperfusion injury.^52^ We noted an increase in serum ALT and AST levels and liver damage in mice after LIRI. However, these changes were attenuated by PMSC treatment and even more effectively by PMSC‐Lin28 treatment. Neutrophil infiltration (MPO staining) was lowest in the LIRI + PMSC‐Lin28 group, followed by the PMSC group and the LIRI group. This suggested that Lin28 can enhance the PMSC‐mediated attenuation of the LIRI‐induced inflammatory response. Moreover, TUNEL staining showed that Lin28 transfection can increase PMSC apoptosis and decrease liver cell apoptosis after LIRI. We consider that overexpression of Lin28 protein can enhance the glycolysis ability of PMSCs by upregulating the PI3K‐Akt‐LDHA pathway, thus enhancing the survival ability of PMSCs under liver ischaemia–reperfusion to achieve a better protective effect against LIRI.

**FIGURE 6:**
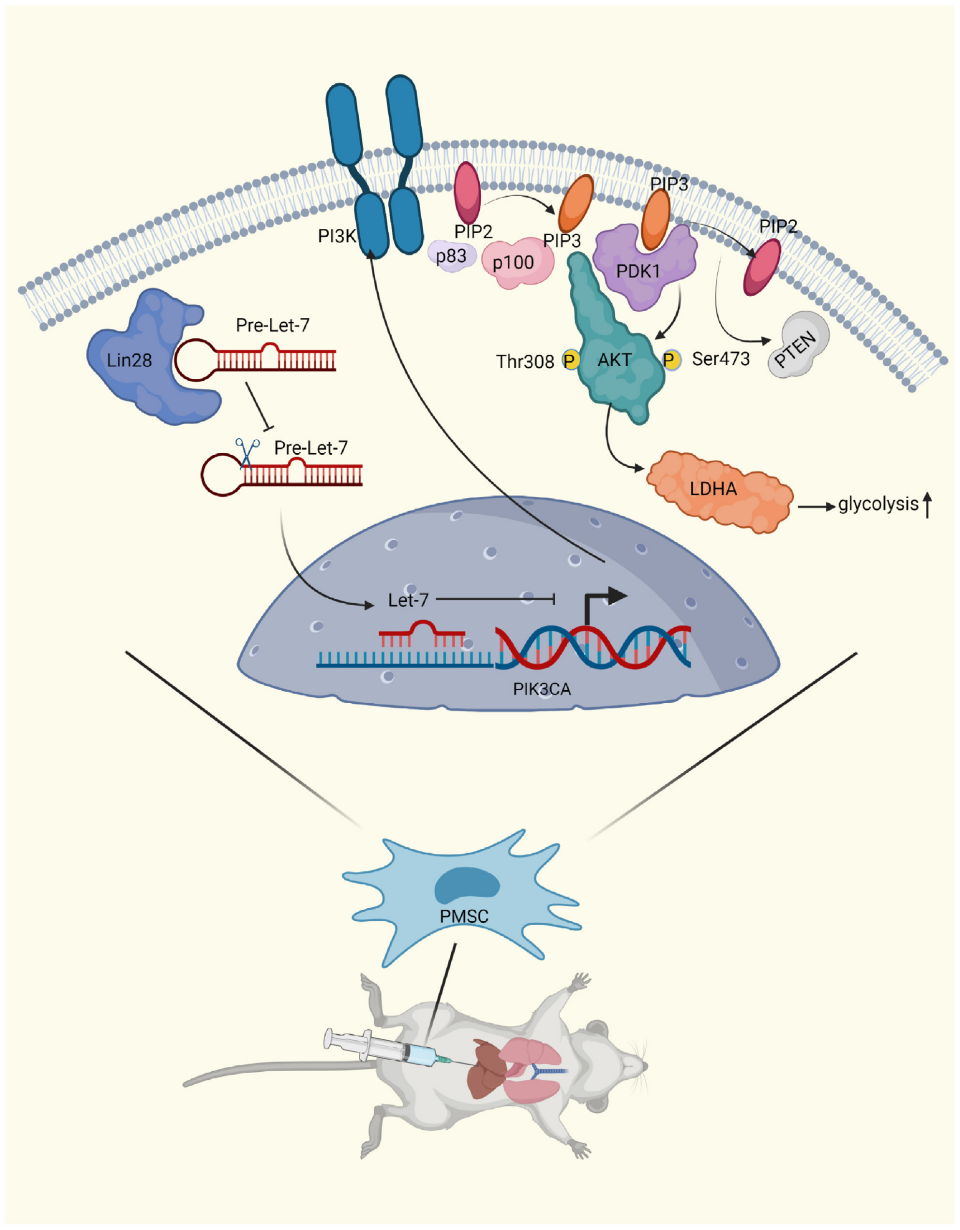
Lin28 increase the protection offered by PMSC against LIRI. First, Lin28 binds to microRNA Let‐7 family precursors, so microRNA Let‐7 family are down‐regulated. Second, microRNA Let‐7 binds to the PIK3CA promoter region to influence its transcription, PI3K phosphorylates AKT and activates AKT, which promotes the expression of LDHA. LDHA increases the glucose metabolism of PMSC and makes PMSC produce more energy in the absence of oxygen, increasing the protection offered by PMSC against LIRI. This diagram were created with BioRender.com.

Although Lin28 is known to activate the PI3K‐Akt pathway,^53^ the mechanism underlying this activation is not well‐understood (Figure 6). We found that the promoter region of the *PIK3CA* gene (which encodes the PI3K α subunit) has a region that can bind to microRNAs from the Let7 family. We speculated that members of the microRNA Let7 family bind to the promoter region of *PIK3CA* and inhibit its transcription. This decreases the expression of PI3K, consistent with our previous results. We aim to explore these mechanisms in subsequent studies.

## 5 CONCLUSIONS

In conclusion, our study shows that Lin28 can improve the glycolytic capacity of PMSCs under hypoxic conditions via the PI3K‐Akt pathway. Therefore, it can promote energy production, even under hypoxia, thus increasing the protection PMSCs offer against LIRI. To our knowledge, our study is the first to show that Lin28 alters glucose metabolism in PMSCs via the PI3K‐Akt pathway, improving LIRI prevention. Despite its limitations, our study provides new insights into the molecular regulation of glycolysis in PMSCs. These insights pave the way for more effective MSC therapies and strategies for LIRI prevention.

## AUTHOR CONTRIBUTIONS

**Xi Zhou:** Data curation (lead); formal analysis (lead); methodology (lead); writing – original draft (lead). **Junbo Li:** Data curation (equal); formal analysis (equal); methodology (equal); visualization (equal). **Jin Wang:** Conceptualization (equal); data curation (equal); investigation (equal); visualization (equal). **Huifang Yang:** Conceptualization (equal); software (equal). **xiaoyun xie:** Conceptualization (equal); writing – review and editing (equal). **Zhishui Chen:** Formal analysis (equal); funding acquisition (equal); writing – review and editing (equal). **Bo Zhang:** Conceptualization (equal); data curation (equal); formal analysis (equal); funding acquisition (equal); investigation (equal); writing – review and editing (equal).

## FUNDING INFORMATION

This work was funded by the National Natural Science Foundation of China (81800580 and 81770652).

## CONFLICT OF INTEREST STATEMENT

The authors have declared that no conflict of interest exists.

## Data Availability

All data included in this study are available upon request by contact with the corresponding author.
